# The Ton Motor

**DOI:** 10.3389/fmicb.2022.852955

**Published:** 2022-04-07

**Authors:** Anna C. Ratliff, Susan K. Buchanan, Herve Celia

**Affiliations:** Laboratory of Molecular Biology, National Institute of Diabetes and Digestive and Kidney Diseases, National Institutes of Health, Bethesda, MD, United States

**Keywords:** TonB, ExbB, ExbD, TonB-dependent transport, proton motive force (pmf), Gram-negative bacteria, membrane proteins, molecular motors

## Abstract

The Ton complex is a molecular motor at the inner membrane of Gram-negative bacteria that uses a proton gradient to apply forces on outer membrane (OM) proteins to permit active transport of nutrients into the periplasmic space. Recently, the structure of the ExbB–ExbD subcomplex was determined in several bacterial species, but the complete structure and stoichiometry of TonB have yet to be determined. The C-terminal end of TonB is known to cross the periplasm and interact with TonB-dependent outer membrane transport proteins with high affinity. Yet despite having significant knowledge of these transport proteins, it is not clear how the Ton motor opens a pathway across the outer membrane for nutrient import. Additionally, the mechanism by which energy is harnessed from the inner membrane subcomplex and transduced to the outer membrane *via* TonB is not well understood. In this review, we will discuss the gaps in the knowledge about the complete structure of the Ton motor complex and the relationship between ion flow used to generate mechanical work at the outer membrane and the nutrient transport process.

## Introduction

**Figure 1:**
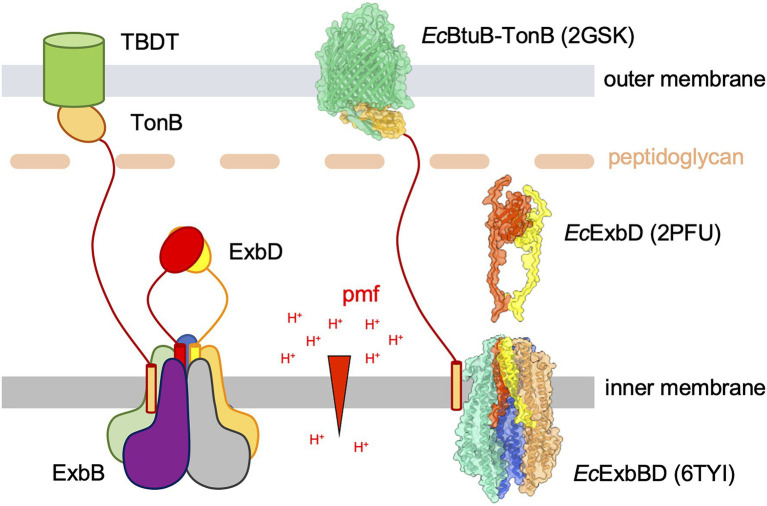
A schematic representation of the Ton uptake system (left) and molecular models of the different components (right). Left panel: the TonB-dependent transporter (TBDT, green cylinder) is anchored in the outer membrane (OM). The ligand binds on the extracellular face of the TBDT and exposes a conserved domain called the TonB box to the periplasmic side. The TonB–ExbB–ExbD (TBD) complex is anchored in the inner membrane (IM) and uses the proton motive force (pmf, proton gradient across the IM, symbolized with the red arrowhead) to generate force and movement. The TBD complex is made of a pentamer of the ExbB subunit (blue, orange, grey, purple, and green) that defines a central pore in which a dimer of ExbD subunits (red and yellow) resides. The TonB subunit (gold) binds at the periphery of the ExbBD subcomplex. The elongated periplasmic domain of TonB allows its C-terminal globular domain to reach the OM and form a stable interaction with the TBDT TonB box. It is hypothesized that the ExbBD subcomplex forms the proton channel and that the energy derived from proton translocation is propagated through the TonB subunit to the TBDT, eventually opening a channel into the TBDT and allowing the bound ligand to diffuse into the periplasm. Right panel: molecular representations of known components of the *Escherichia coli* Ton system. The structural models are shown with ribbons and molecular surfaces. The color coding is the same than for the schematic view on the left. The TM and flexible periplasmic domain on the *Ec*TonB subunit are not known and shown as in the schematic representation. The crystallographic structure of *Ec*BtuB (green) in complex with the *Ec*TonB periplasmic domain (gold) is represented in the OM (pdb 2GSK; Shultis et al., 2006). Two models of the NMR structure of the *Ec*ExbD periplasmic domains are shown in red and yellow (pdb 2PFU; Garcia-Herrero et al., 2007). The cryo-EM structure of the *Ec*ExbBD complex is shown in the IM (pdb 6TYI; Celia et al., 2019). The grey and purple ExbB subunits are not represented in order to show the ExbD TM domains. Molecular graphics have been performed with UCSF ChimeraX (Pettersen et al., 2021).

The bacterial outer membrane (OM) is the first line of defense for Gram-negative bacteria against its environment. The outer membrane forms a resistant barrier against toxins and environmental threats yet must allow a variety of substances to cross without compromising the membrane. The OM can be permeated through outer membrane proteins that facilitate the diffusion of small molecules and nutrients into the periplasm (Nikaido, 2003; Vergalli et al., 2019). The OM lacks a hydrolysable energy source or an electrochemical gradient; thus, some nutrients are large or at low concentrations are actively transported across the OM with the aid of the Ton complex. Ton is a multi-subunit membrane protein complex (TonB–ExbB–ExbD) that uses the proton gradient across the inner membrane as its energy source (Figure 1). At the inner membrane, ExbB and ExbD harness the proton motive force (pmf) and transfer it to the TonB subunit. TonB is anchored to the inner membrane by a single N-terminal transmembrane helix and has an ordered C-terminal domain linked by a central proline-rich periplasmic domain (Kohler et al., 2010). This linker is long enough to span the whole periplasmic space, allowing the TonB C-terminal domain to reach the OM and interact with TonB-dependent transporters (TBDTs), providing energy for nutrient import. In this review, we will discuss the gaps in the knowledge about the structure of the complete Ton motor complex (TonB–ExbB–ExbD, or TBD), as well as the molecular interactions and mechanism by which the complex uses the pmf at the inner membrane to generate mechanical work at the outer membrane for the transport of nutrients.

## Overview of the Ton Uptake System

TonB-dependent transporters are comprised of a 22-stranded ß-barrel C-terminal domain with a N-terminal plug domain inserted into the interior of the barrel (Noinaj et al., 2010). TBDTs import a range of small molecules and nutrients based on the specificity of the transporter, which include iron-siderophores, divalent metals, carbohydrates, cobalamin, and peptides (Schauer et al., 2008; Noinaj et al., 2010; Calmettes et al., 2015; Madej et al., 2020). Upon ligand binding on the extracellular face of the TBDT, conformational changes are induced, exposing the TonB box, a short, conserved N-terminal sequence, to the periplasm. The C-terminal periplasmic domain of the TonB subunit then interacts with the TBDT TonB box, forming a stable complex that physically connects the TBDT to the inner membrane with measured affinity up to tens of nanomolar (Freed et al., 2013; Sarver et al., 2018; Josts et al., 2019). What happens after the formation of the TBDT and TonB complex is widely unknown, but it is hypothesized that the energy from the pmf is transmitted to TonB and used to alter the conformation of the TBDT, eventually opening a channel in the TBDT.

## Structures of the Ton Complex

Over the past decade the stoichiometry of ExbB and ExbD has been highly disputed, but recent reports strongly support that the ExbBD subcomplex has a 5:2 ratio with ExbB forming a pentameric hydrophobic central pore encircling a dimer of ExbD single helices (Figure 1; Celia et al., 2020). The first high-resolution structure of the *Ec*ExbBD subcomplex showed *Ec*ExbB as a pentameric structure (Celia et al., 2016). Mass spectrometry experiments performed on *Escherichia coli*-native membranes further supported the pentameric nature of *Ec*ExbB (Chorev et al., 2018). Cryo-EM single-particle analysis (SPA) was also used to determine high-resolution structures of the ExbBD complexes from *E. coli*, *Pseudomonas savastanoi*, and *Serratia marcescens*, all revealing a 5:2 ratio (Celia et al., 2019; Deme et al., 2020; Biou et al., 2021).

MotAB and PomAB are the motor complexes that power the rotation of the flagellum, using the proton or sodium gradient, respectively. Both share extensive homology with ExbBD and are believed to derive from a common ancestor (Marmon, 2013; Lai et al., 2020).

MotAB, PomAB, and ExbBD share a high level of conservation in the transmembrane helices that form the central hydrophobic pore of ExbB/MotA/PomA. Recently published cryo-EM structures of MotAB and PomAB also confirmed 5:2 ratio of these motor complexes (Deme et al., 2020; Santiveri et al., 2020). For all these structures of ExbBD and MotAB determined by cryo-EM, the periplasmic domains of ExbD and MotB were not visible because of their high flexibility.

TonB appears to cycle through binding and release from TBDTs, sometimes referred to as the TonB energization cycle (Larsen et al., 1999; Kaserer et al., 2008; Gresock et al., 2015). While the structure of the *Ec*ExbBD subcomplex is now established, a detailed molecular description of a full TBD complex is not yet available. A cryo-EM map of the full *P. savastanoi PS*ExbB complex has been reported (EMD-10897; Deme et al., 2020). The 3.8 Å resolution structure clearly shows the *Ps*ExbB and *Ps*ExbD subunits, with an additional rod-like structure that likely corresponds to a single TMH of a TonB subunit. The density traverses the micelle on the exterior of the *Ps*ExbB complex and packs against a region of TM1 of one *Ps*ExbB, in a location predicted by analysis of covariance and close to the cytoplasmic leaflet of the membrane (Deme et al., 2020). The lower density and resolution of the TonB TMH in the cryo-EM map were attributed to a partial dissociation of TonB from ExbBD upon freezing. Cryo-EM SPA of the *E. coli Ec*TBD complex brought similar observations, showing the TonB TMH in a similar orientation compared to *Ec*ExbBD, and interacting with part of TM1 of *Ec*ExbB (H.C. unpublished results). The precise oligomeric state of TonB in the TBD complex is still not clearly known and needs to be further investigated.

Numerous studies have focused on the molecular interactions between the periplasmic domains of ExbD and TonB. The C-terminal domain of TonB can form a dimer *in vivo* (Sauter et al., 2003). The physiological importance of this dimer is not well-established, but it might be involved in the binding to the peptidoglycan (PG) layer, therefore localizing the TonB C-terminal domain close to the OM (Ghosh and Postle, 2004, 2005; Kaserer et al., 2008; Postle et al., 2010). The C-terminal folded domain of *Ec*ExbD is a dimer in the *Ec*ExbBD complex (Figure 1; Gresock et al., 2015; Celia et al., 2016). The *Ec*ExbD dimerization interface might be altered during the energization cycle as some *Ec*ExbD monomers were found to be interacting with the TonB C-terminal domain in a pmf-dependent fashion (Ollis and Postle, 2011, 2012). This network of interactions likely reflects a dynamic interplay between ExbD and TonB during the energization process.

Both TonB and ExbD have a flexible periplasmic linker between their TM and C-terminal folded domains (Figure 1). Recent studies have highlighted the importance of the disordered, periplasmic linker domain of *Ec*ExbD. A conserved motif just upstream of the TM domain of *E*cExbD, V45, V47, L49, and P50 was found to be required for Ton function (Kopp and Postle, 2020). The TonB periplasmic linker is long enough to allow the C-terminal folded domain of TonB to reach the TBDTs in the OM. Most TonB sequences exhibit a conserved proline-rich domain in the linker region that is suspected to adopt a poly-proline type II helical rod conformation, conferring rigidity to the linker (Chu et al., 2007; Kohler et al., 2010). However the presence of this poly-proline-rich domain was found nonessential for energy transduction (Larsen et al., 1994).

## ExbBD and MotAB Share a Similar Proton Channel

The Ton, Tol, and Mot complexes use the pmf to generate movement and share extensive homology (Cascales et al., 2001; Marmon, 2013; Ratliff et al., 2021). Tol is involved in the regulation of the OM integrity and cell division through interaction with the OM-associated Pal-TolB protein complex. Mot is powering the flagellum rotation. The Ton and Tol complexes are the most closely related: the TonB–ExbB–ExbD and TolA–TolQ–TolR subunits have the same topology, are highly homologous, and cross-complementation between TolA and ExbBD, and TonB and TolQR, has been observed (Braun and Herrmann, 1993; Lloubes et al., 2012). While there is no reported structure of TolQR, it is expected to be very similar to ExbBD (for more information on the Tol system, see Szczepaniak et al., 2020; and the dedicated review in this issue of *Frontiers in Microbiology*).

The MotAB complex is homologous to ExbBD/TolQR but lacks a TonB/TolA-like subunit. It associates with the motility apparatus and uses the pmf to generate torque, driving the rotation of the flagellum (Minamino and Imada, 2015; Lai et al., 2020). Like ExbD and TolR, MotB has a single TM domain, followed by a flexible periplasmic linker and a folded C-terminal domain. Between the TM and the periplasmic linker, MotB has a conserved sequence that associates with MotA and acts as a plug that prevents the flux of protons through the complex (Hosking et al., 2006). It is believed that upon association of the MotAB complex to the flagellar apparatus, the C-terminal domains of MotB bind the PG and the plug domains dissociate from MotA, allowing the flux of proton and torque generation (Kojima et al., 2009, 2018; O'Neill et al., 2011).

**Figure 2:**
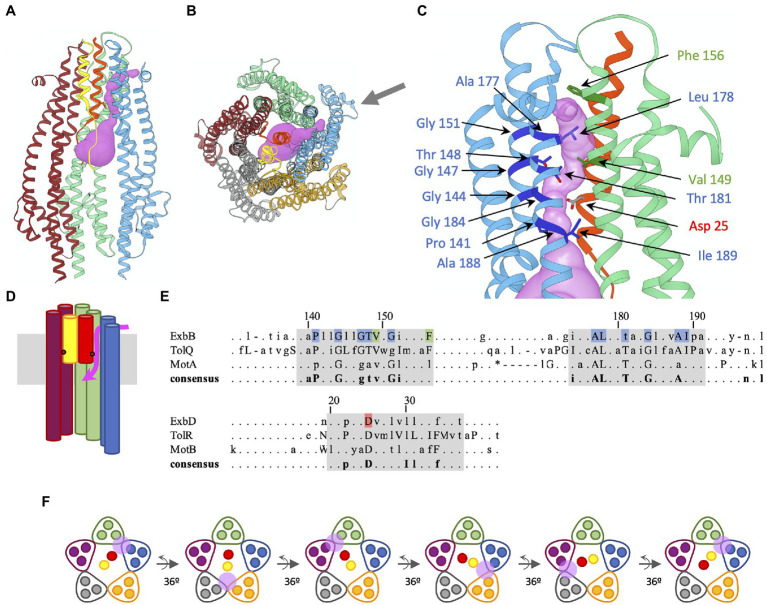
Predicted proton channel in *Ec*ExbBD, sequence conservation of residues lining the channel, and rotary model of the ExbD TMs in the ExbB pentamer. The color coding of the different subunits is the same as in Figure 1. **(A)** Ribbon representation of the *Ec*ExbBD complex (pdb 6TYI), with the predicted proton channel represented as pink isosurface. For clarity, two ExbB subunits (grey and orange) have been omitted to reveal the interior of the complex. The blue and green *Ec*ExbB subunits are involved in the channel formation. The TM helices of *Ec*ExbD are colored yellow and red, with the side chains of the essential Asp25 represented as ball and sticks. The red helix is involved in the formation of the channel. **(B)** Same ribbon representation as **(A)**, but viewed from the periplasm, all the *Ec*ExbBD subunits are shown. The online version of Mole 2.5 (https://mole.upol.cz/; Pravda et al., 2018) was used to probe for cavities and channels in the *Ec*ExbBD structure. The same parameters reported for the *Cj*MotAB channel (Santiveri et al., 2020) were used: 1 Å radius bottleneck, and omission of Leu178 side chain for the calculation. The black arrow shows the direction of viewing for **(C)**. **(C)** Enlarged view of the channel. Only the *Ec*ExbB and *Ec*ExbD subunits involved in the channel formation are shown (blue, green, and red). The most conserved residues in the consensus sequence shown in **(E)** are highlighted in darker colors and side chains are shown as ball and stick. **(D)** Schematic representation of **(A)**, showing the path of the channel. Only the TMs 2 and 3 of the blue, green, and purple *Ec*ExbB subunits are shown. The approximate location of the conserved Asp on *Ec*ExbD TM is shown with red dots. The membrane is shown in grey. The channel is symbolized in pink. It opens between the blue and green *Ec*ExbB subunits on the periplasmic side, connects to the Asp25 of the red *Ec*ExbD TM, and opens on the cytoplasmic cavity of the *Ec*ExbB pentamer. **(E)** Consensus from the multiple sequence alignment of ExbB/TolQ/MotA last two TM domains and ExbD/TolR/MotB TM domain, adapted from Figure 4 from Cascales et al. (2001). Lowercase letters represent residues present in the 60% consensus and uppercase letters for residues in the 90% consensus. Gaps are marked “–” and “*” when present in the 90 and 60% consensus, respectively (Cascales et al., 2001). The numbering corresponds to the *E. coli* sequences of ExbB and ExbD. The highlighted residues in blue, green, and red are the ones shown with arrows on **(C)**. They all are in the 90% consensus range, except for Thr181. The regions highlighted in grey correspond to the last two TMs of ExbB, TolQ, and MotA, and the single TM of ExbD, TolR, and MotB. **(F)** Rotary model of ExbBD. The view is the same as in **(B)** and shows a schematic slice of the TM domains of ExbB and ExbD. The positions of the proton channel are shown with the pink circle. The cycle starts with the channel opening between the green and blue ExbB subunits. The proton travels to the conserved Asp on the red ExbD TM, inducing a conformational change resulting in the rotation of the two ExbD TMs by 36°. The conformational changes lead to the closure of the channel between the green and blue ExbB subunits, while a new channel opens between the grey and orange ExbB subunits. A second proton now travels to the Asp on the yellow ExbD TM, resulting in a new rotation of 36°. The channel between the grey and orange ExbB subunits closes, while a new channel opens between the purple and green subunits, allowing a third proton to travel to the Asp on the red ExbD TM. The rotation can proceed as long as the channels are in the open state. Molecular graphics have been performed with UCSF Chimera (Pettersen et al., 2004).

Several high-resolution cryo-EM structures of MotAB have been reported, and all show the same 5:2 architecture as ExbBD (Celia et al., 2019; Deme et al., 2020; Santiveri et al., 2020; Biou et al., 2021). The transmembrane region that forms the pore encircling the ExbD/MotB TM helices is particularly conserved between MotAB and ExbBD (Deme et al., 2020; Ratliff et al., 2021). The Taylor lab reported three distinct structures of the *Campylobacter jejuni Cj*MotAB complex: *Cj*MotAB in the plugged state, a construct lacking the *Cj*MotB plug domain, and a construct lacking the *Cj*MotB plug domain plus a substitution of the conserved essential Asp22 residue in the *Cj*MotB TM domain into Asn, so as to mimic the protonated state of Asp22 (Santiveri et al., 2020). Using Mole 2.5, a software that detects tunnels and cavities in macromolecules, a potential proton channel that connects the conserved Asp22 on *Cj*MotB to the periplasm was revealed (Pravda et al., 2018; Santiveri et al., 2020). Close to the opening on the periplasmic side, the side chain of residue Phe186 on *Cj*MotA TM4 was found in two different conformations, eventually acting as a gate that would open and close the proton channel to the periplasm. Sequence alignments show that the Phe186 is a conserved residue with a bulky side chain, mostly appearing as Leu in the consensus conserved sequence of MotA, ExbB, and TolQ (see Figure 2E; Cascales et al., 2001).

We used a similar approach to probe the structure of *Ec*ExbBD (PDB 6TYI) for potential channels. Using the same parameters for Mole 2.5 and omitting in the calculation the side chain of Leu178 in TM3 of *Ec*ExbB (equivalent of Phe186 in TM4 of *Cj*MotA), a channel was found that connects the periplasmic side of *Ec*ExbBD and the essential Asp25 on *Ec*ExbD TM that opens to the cytoplasmic cavity (Figures 2A–D). The channel opens between two *Ec*ExbB subunits (blue and green represented chains) on the periplasmic side, close to Phe156 (Figures 2C,D). In the configuration shown in Figure 2C, the channel is occluded by Leu178 side chain. As for Phe186 of *Cj*MotA, Leu178 could act as a gate that would modulate the opening of the proton channel. Figure 2E shows the consensus sequence resulting from multiple alignments of ExbB/TolQ/MotA and ExbD/TolR/MotB (Cascales et al., 2001). Highlighted in blue and green (*Ec*ExbB TM2-3) and red (*Ec*ExbD TM) are the residues that are lining up the channel found in the *Ec*ExbBD structure.

It is noteworthy that most of these conserved residues apparently involved in the channel formation are highly conserved among ExbB, TolQ, and MotA as shown in the consensus sequence Figure 2E. The channels for *Ec*ExbBD and *Cj*MotAB are remarkably similar, reflecting the high homology of the two systems, and likely a similar usage of proton translocation to generate movement.

Rotary models have been proposed for the MotAB complex to harness the pmf, which are discussed elsewhere in this issue of *Frontiers in Microbiology*. These models suggest that the MotA pentamer rotates around the MotB dimer at 36° increments for each proton translocated (Deme et al., 2020; Santiveri et al., 2020). While it is not yet clear what prompts the movement of MotA following the protonation/deprotonation of the conserved Asp on MotB, it is likely that a rotation mechanism is responsible for torque generation. Since the MotAB and ExbBD complexes are highly homologous, it is expected that the use of the pmf is the same and that the ExbB pentamer rotates around the ExbD dimer as well during proton translocation.

Based on these models, we propose a rotary model in which the ExbD TMs rotate by increments of 36° for each proton translocated (Figure 2F). It is hypothesized that the proton travels through the open channel from the periplasm to the highly conserved Asp on ExbD TM, inducing a change of conformation of protonated Asp that would drive the power stroke, resulting in the rotation of the two ExbD TMs by 36°. The change of conformation would close the channel on the side of the protonated Asp, while a new channel would open on the opposite ExbD TM, allowing a new cycle to take place. The rotation would then continue as long as the channels are in the open state. Is it not known how the proton channel opens, and how the open state is regulated, but it likely depends on the TonB subunit.

## Wrap and Pull Model Mechanism

Several models for the mechanism of action of Ton have been proposed. They all rely on the association of the TonB C-terminal domain with the TBDT TonB box, and the application of force to displace or alter the conformation of the TBDT plug domain, either through rotation or pulling of TonB (Chimento et al., 2005; Klebba, 2016).

In the *pulling model*, the TonB C-terminal domain bound to the TBDT is pulled into the periplasm by the ExbBD complex and gradually unfolds the TBDT plug domain, eventually opening a channel large enough to allow the bound nutrient to diffuse toward the periplasm (Chimento et al., 2005). Molecular simulations using the BtuB TBDT in complex with TonB show that the interaction between BtuB and TonB is strong enough to sustain a pulling force perpendicular to the OM plane and would partially unfold the BtuB plug domain (Gumbart et al., 2007). This mechanism is supported by *in vitro* single-molecule force spectroscopy experiments on the BtuB/TonB and FhuA/TonB complexes, providing direct evidence that the interaction between TonB and the TonB box is strong and can withstand the amount of force needed to unfold half of the TBDT plug domain before dissociation occurs (Hickman et al., 2017).

Some bacteriocins bind TBDTs and hijack the Ton system to gain access and kill bacteria with high efficiency (Atanaskovic and Kleanthous, 2019). The bacteriocin pyocin S2 has its own TonB box motif and has been shown to translocate through the *P. aeruginosa* iron transporter FpvAI (White et al., 2017). Pyocin S2 binds FpvAI at the same binding site than the natural pyoverdin-iron siderophore and initiates the partial unfolding of the plug domain through interaction of the FpvA TonB box with TonB. The TonB box motif of pyocin S2 is then presented to TonB through the channel created, and the force exerted by the Ton system eventually unfolds the pyocin and drags it into the periplasm (White et al., 2017). This work represents the first time the translocation of a bacteriocin through a TBDT was shown, and the mechanism is in good agreement with the pulling model.

**Figure 3:**
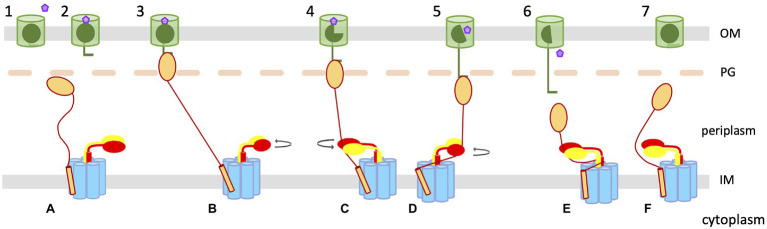
Wrap and pull mechanistic model. The TBDT in the OM is represented with a green cylinder, with the plug domain and TonB box in dark green. The ligand to be transported is represented as a pink pentagon. TonB–ExbB–ExbD in the inner membrane are shown in gold (TonB), blue (ExbB), and red and yellow (ExbD), respectively. (1) The TBDT binds to its ligand inducing the exposure of the TonB box (2). **(A)** The TBD complex is in its resting state. The TonB C-terminal domain binds to the exposed TonB box of the loaded TBDT (3; **B**) tethering TonB to the OM, and somehow inducing a conformational change in ExbBD that opens the proton channel, triggering the rotation of the ExbD dimer **(B)**. (4; **C**) The rotation of ExbD leads to direct contact of ExbD with the TonB periplasmic linker and starts to pull on the C-terminal domain of TonB bound to the TBDT. **(D)** While the rotation of ExbD continues, the TonB linker wraps around ExbD, pulling further on the TonB–TBDT complex, partially unfolding the TBDT plug domain (5). (6) The unfolding of the plug domain reaches a point where the opening of the channel allows the ligand to diffuse to the periplasm, and the force necessary to unfold the rest of the plug is greater than the force necessary to maintain the interaction between TonB and the TonB box. TonB then dissociates from the TBDT **(E)**, releasing the tension on the TonB periplasmic linker and reverting the ExbBD complex to the closed state. (7) The plug domain folds back into the TBDT barrel; and **(F)** the TBD complex goes back to its resting state.

To reconciliate the pulling model with an eventual rotation of ExbBD, we propose a model in which the rotation of the ExbD dimer leads to the wrapping of the TonB linker around ExbD, leading to the pulling of the TonB C-terminal domain bound to the TBDT TonB box (Figure 3). In this model, the tethering of TonB to the OM, through the interaction between the TonB C-terminal domain and the TBDT TonB box, induces a conformational change in ExbBD that opens the proton channel. The translocation of protons through ExbBD leads to the rotation of the ExbD dimer within the ExbB pentamer (Figure 2F) and the associated TonB. The periplasmic domain of ExbD comes into contact with the TonB periplasmic linker, which starts to wrap around ExbD while the rotation proceeds. The wrapping of the two proteins leads to a pulling motion on the C-terminal domain of TonB, which gradually unfolds the plug domain of the TBDT. With the unfolding of the plug domain proceeding, a channel through the TBDT gradually opens, allowing the ligand to move into the periplasm. At some point, the force necessary to further unfold the plug domain is greater than the interaction between the TonB box and TonB, causing TonB to dissociate from the TBDT. TonB is no longer tethered to the OM, causing the proton channel to close. The TonB box and plug domains fold back into the TBDT barrel, and the TonB linker unwraps, reverting both the TBDT and TBD complex to their resting states.

Further work is needed to better understand how the Ton system functions. The high-resolution structures of ExbBD and MotAB have provided insights on how the pmf is used by these protein complexes. The high structural and sequence homologies between the two systems suggest that they share the same mechanism, and any new information gathered on one system will likely translate to the other. Some of the important unanswered questions for the Ton system are: how is the signal of the binding of TonB to the TBDT TonB box at the OM transmitted to TBD at the IM? what are the molecular events that open the proton channel in ExbBD?; and how is the potential rotation of ExbB and ExbD transferred to TonB? A combination of *in vivo* and *in vitro* approaches is needed to further understand this multicompartment system.

## Author Contributions

AR, SB, and HC contributed to writing the manuscript and editing. HC prepared figures. All authors contributed to the article and approved the submitted version.

## Funding

AR, HC, and SB are supported by the Intramural Research Program of the NIH, National Institute of Diabetes and Digestive and Kidney Diseases (NIDDK).

## Conflict of Interest

The authors declare that the research was conducted in the absence of any commercial or financial relationships that could be construed as a potential conflict of interest.

## Publisher’s Note

All claims expressed in this article are solely those of the authors and do not necessarily represent those of their affiliated organizations, or those of the publisher, the editors and the reviewers. Any product that may be evaluated in this article, or claim that may be made by its manufacturer, is not guaranteed or endorsed by the publisher.
